# Efficacy of Chitosan-Based Dressing for Control of Bleeding in Excisional Wounds

**Published:** 2018-03-10

**Authors:** Anne-Heloise Stricker-Krongrad, Zahra Alikhassy, Nicolette Matsangos, Raul Sebastian, Guy Marti, Frank Lay, John W. Harmon

**Affiliations:** ^a^Section of Surgical Sciences, Department of Surgery, John Hopkins Bayview Medical Center, John Hopkins University School of Medicine, Baltimore, Md; ^b^Department of Surgery, Johns Hopkins School of Medicine, Baltimore, Md; ^c^Department of Surgery Clinique Saint-Jean-l'Ermitage Hospital, Melun, France

**Keywords:** chitosan, homeostatic, dressing, anticoagulants, debridement

## Abstract

**Introduction:** Excessive bleeding is a complication of wound debridement in patients receiving anticoagulation treatment. Chitosan is a linear, positively charged polysaccharide that has potential as a hemostatic topical dressing. This study examined the hemostatic efficacy of the chitosan based Opticell dressing (Medline Industries, Chicago, Ill) in heparinized rats with excisional wounds mimicking debridement. **Methods:** Three paired 12-mm excisional wounds were created on the dorsum of 600-g Sprague-Dawley rats 2 hours after intraperitoneal injection of heparin 800 IU/kg. Opticell or gauze dressings were applied with 3 seconds of gentle pressure. **Results:** *Total Bleeding*: The dressings were left in place until cessation of bleeding. Ten minutes was enough time for complete bleeding cessation in both groups. Gauze and Opticell were weighed before and after bleeding cessation, with the difference representing blood loss. Total blood loss was 627 ± 47 mg/10 min with the standard gauze, but 247 ± 47 mg/10 min with Opticell (*P* = .002 Mann-Whitney). N = 6 wounds per group. **Rate of Bleeding:** Gauze and Opticell dressings were removed and instantly replaced with 3 seconds of gentle pressure every minute until bleeding cessation. The removed dressings were weighed before and after application. There was less bleeding in the Opticell group at minutes 1, 2, and 3. Gauze: 183 ± 40, 140 ± 30, and 109 ± 15 mg/min vs Opticell: 91 ± 17, 54 ± 8, and 57 ± 11 mg/min). Analysis of variance, Tukey's test, *P* < .05. N = 12 wounds per group. **Conclusion:** Topical application of Opticell dressing with chitosan has hemostatic effects that could be a useful tool to control bleeding associated with wound debridement.

Wound healing involves several overlapping phases: hemostasis, inflammation, proliferation, and remodeling.^1^ Immediately after injury, hemostasis produces a blood clot that covers the wound.^2^ During the hemostasis phase, after activation of platelets, a much-regulated panel of growth factors, cytokines, and chemoattractants are released from injured tissue.

Chitosan is a promising hemostatic agent because it can adhere to red blood cells and encourages platelets to adhere, activate, and aggregate at the site of vascular injury.^3^ Chitosan is a linear, positively charged polysaccharide derived from chitin, a compound of natural origin obtained from the shell of crabs and shellfish, which has potential as a hemostatic topical dressing.^4^ Because of chitosan biodegradability and its biological properties, such as hemostatic activity, antibacterial activity, and ability to accelerate wound healing, it is being used in many medical devices and health care products.^5^ Chitosan-based dressing has been reported to accelerate hemostasis even in the presence of coagulopathy.^6^^,^^7^

Prolonged primary anticoagulation could impair the healing process.^8^ A minor wound could be a daily challenge for patients who use anticoagulants and antiplatelet drugs. Standard gauze dressings and direct pressure are often time-intensive for controlling hemorrhage for these patients.

There is increasing interest in the pharmacologic control of hemorrhage. Much of this focuses on massive hemorrhage. Topical treatment of massive hemorrhage includes QuiK Clot (Walingford, Conn) and various thrombin-based products. Factor VII parenteral treatment is being explored for uncontrolled bleeding but is so powerful that it carries a risk for causing cerebral artery thrombosis.^9^ The inhibitor of fibrinolysis tranexamic acid was shown to be effective in massive hemorrhage in the CRASH2 trial but is still undergoing review to discover its appropriate role in hemostasis.^10^

The current study described in this report was designed with the modest aim of exploring the potential of a chitosan-based topical dressing for reducing hemorrhage from an excisional wound in the setting of heparin anticoagulation. This scenario is clinically relevant, as it is similar to the commonly performed wound debridement for patients receiving anticoagulation therapy.

## CLINICAL PROBLEM ADDRESSED

The aim of this study was to determine the efficacy of a chitosan-based dressing for hemostasis using an animal model to mimic clinical wound debridement for patients taking anticoagulants.

## MATERIALS AND METHODS

The Johns Hopkins Animal Care and Use Committee approved this study. Male Sprague-Dawley rats weighing between 630 and 800 g were purchased from Charles River Laboratories, Frederick, Md.

To determine the efficacy of chitosan-based dressing on debridement of wounds for patients taking anticoagulants, chitosan dressing and woven gauze were compared in a rat model with impaired coagulation. Heparin, an anticoagulant, was used to stimulate blood loss in excisional wounds using a standard rat protocol.^11^

The chitosan dressing used in these experiments is made by Medline (Chicago, Ill). Chitin is an abundant, naturally occurring structural polysaccharide that is the main component of nonvertebrate exoskeleton. Chitin is difficult to use as a biomaterial without modification. For the dressing used in these experiments, chitosan was spun into fibers in order to result in a soft, absorbent material suitable for a wound dressing.

### Determining timing and dosage

**Figure 1:**
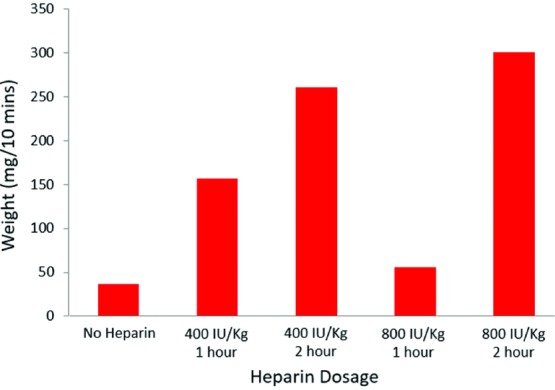
Comparison of blood loss of 800 IU/kg of heparin versus 400 IU/kg of heparin versus no heparin.

The initial step was to determine the suitable dosage of heparin to produce significant bleeding from excisional wounds. Heparin was administered according to a previously published protocol.^11^ Two 12-mm circular full-thickness wounds were made on the dorsal dermis of 5 rats. Five dosing regimens were compared. The regimens consisted of 400 IU/kg of heparin administered 1 hour prior to surgical dermal excision, 400 IU/kg of heparin administered 2 hours prior to surgical dermal excision, 800 IU/kg of heparin administered 1 hour prior to surgical dermal excision, 800 IU/kg of heparin administered 2 hours prior to surgical dermal excision, and, finally, no heparin administered. Gauze dressings were applied. The dressings were weighed before and after application (Fig 1). Blood loss was quantified by the difference in weight before and after blood cessation. The 800 IU/kg dosage at 2 hours was chosen, as it produced significant bleeding. In addition, this highest dosage was considered to be an appropriate challenge for the chitosan dressing.

**Figure 2:**
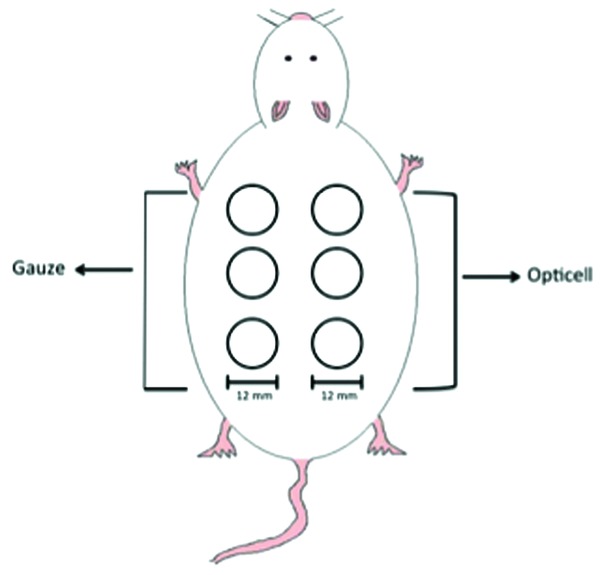
Visual representation of full-thickness wounds on rat model for part one 1 and part 2.

**Figure 3:**
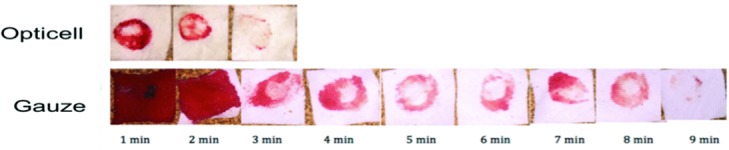
Photographic examples showing comparison of blood loss of gauze versus Opticell.

For the comparison of chitosan dressing and the woven gauze dressing, all animals were administered 800 IU/kg of heparin via intraperitoneal route 2 hours prior to surgical dermal excision. Animals were housed in single cages. Six paired, 12-mm circular, full-thickness cutaneous wounds, 3 wounds on either side, were made on the dorsal dermis of each rat (Figs 2 and 3).

### Dynamic assessment of bleeding (part 1)

For this part, 4 rats were used. Chitosan dressing was applied on one side (3 wounds) and gauze on the other (3 wounds). Gauze and the chitosan dressing were removed and instantly replaced using consistent, brief, 3-second gentle pressure every minute until bleeding cessation. The pressure applied over skin was the same for both sides. The dressings were weighed before and after application. The increase in weight was quantified as blood loss per minute (mg/min).

### Total bleeding evaluation (part 2)

Two rats were used in this part. Six paired, 12-mm circular, full-thickness wounds, 3 wounds on either side, were made on the dorsal dermis of each rat. Chitosan dressing was applied on one side and woven gauze on the other. They were left in place until cessation of bleeding. Complete bleeding cessation occurred after 10 minutes in both groups. Gauze and chitosan dressing were weighed before and after bleeding cessation, with the difference representing blood loss. Blood loss was expressed in mg/10 min.

## RESULTS

### Determination of heparin dosing

As shown in Figure 1, the animal administered 800 IU/kg of heparin 2 hours prior to excisional wound bled 300 mg/10 min. Bleeding seen with the other dosages were as follows: 800 IU/kg administration for 1 hour was 56 mg/10 min, 400 IU/kg administration for 2 hours was 47 mg/10 min, and 400 IU/kg administration for 1 hour was 157 mg/10 min. These results indicate that 800 IU/kg of heparin 2 hours prior to excisional wound stimulates a sufficient amount of blood loss to challenge the hemostatic capability of chitosan dressing versus standard gauze.

### Dynamic assessment of bleeding

**Figure 4:**
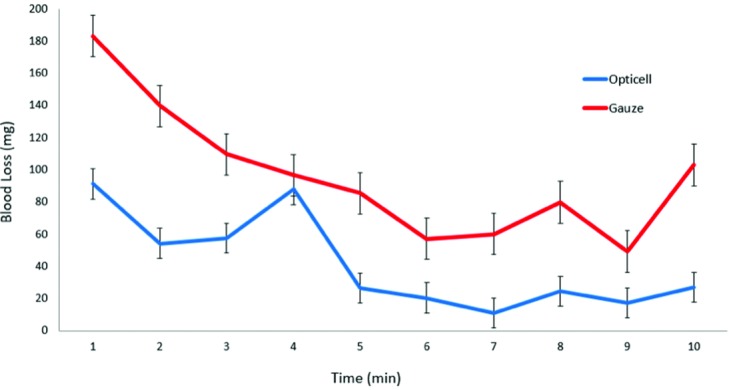
Average blood loss after 2 hours of heparin administration in 4 male retired breeder rats.

**Figure 5:**
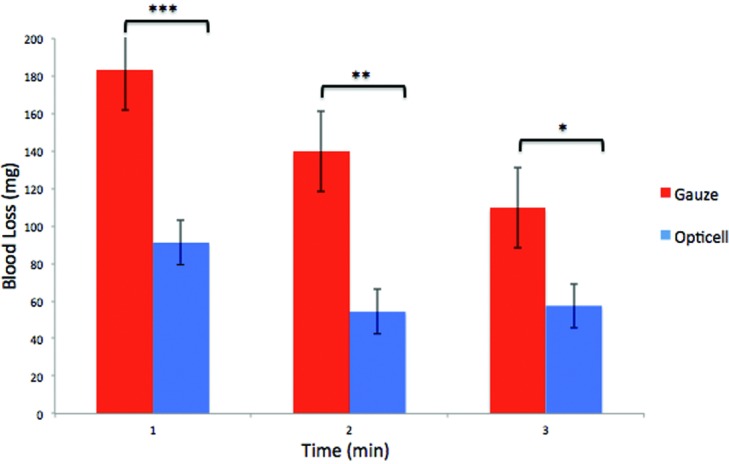
Significant differences between Opticell and gauze (***P = .002, **P = .004, *P = .045).

All animals for part 1 and part 2 were housed in single cages for 2 hours after administration of heparin. All six 12-mm circular full-thickness wounds were created after the 2-hour period. The dressings were weighed before and after in contact with the bleeding wound. Blood loss was assessed immediately after every minute until bleeding cessation. The average blood loss in the chitosan dressing group, in the consecutive span of 10 minutes, was 91, 54, 58, 88, 27, 20, 11, 25, 17, and 27 mg. The average blood loss in the gauze group was 183, 140, 110, 97, 85, 57, 60, 80, 49, and 103 mg (Fig 4). On the basis of the average blood loss values, analysis of variance with the Tukey posttest was used to evaluate differences in mean blood loss for significance. Significant differences in blood loss were seen with the chitosan dressing at minutes 1, 2 and 3 (***P = .002, **P = .004, *P = .045) (Fig 5). The results from part 1 indicate that the efficacy of chitosan-based dressing for bleeding cessation was superior to the standard gauze.

### Total bleeding evaluation

**Figure 6:**
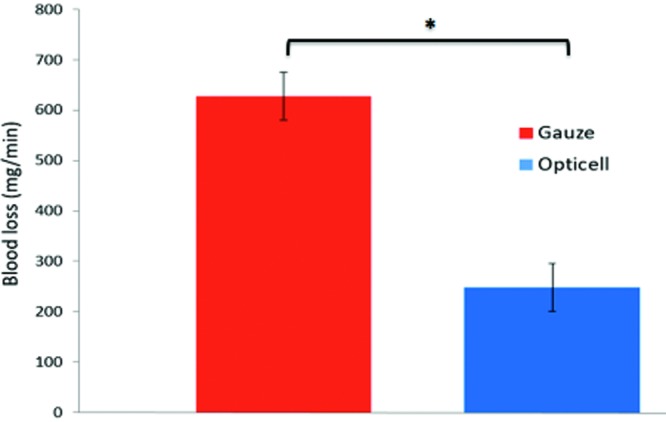
Difference between Opticell and gauze (*P = .002).

Complete bleeding cessation in both groups was noted after 10 minutes. The dressings were weighed before and after application. The difference between the weights before and after application represents blood loss. The results for part 2 showed that postsurgery, the total blood loss was 247 ± 47 mg/10 min in the chitosan dressing group versus 627 ± 47 mg/10 min in the gauze group. The lower blood loss over 10 minutes indicates more effective hemostatic properties of the chitosan dressing compared with the standard woven gauze (Fig 6).

## DISCUSSION

Uncontrolled hemorrhage is a potential risk factor for those taking anticoagulants. During wound debridement, excessive and prolonged bleeding is disturbing for patients, prolongs the efforts of the wound care providers, and may interfere with the healing process. An easy-to-use and effective hemostatic dressing is an attractive solution to this problem.

Chitosan is known to have hemostasis properties such as promotion of platelet adhesion, platelet aggregation, and platelet activation.^12^ The aim of this study was to utilize an animal model to determine the potential efficacy of a chitosan-based dressing, specifically Opticell, to promote hemostasis after debridement of wounds for patients taking anticoagulants.

### Mode of action of heparin

Because of prolonged bleeding time in patients using anticoagulants, even a small trivial wound could be daily concern. Heparin is a classic anticoagulant that inhibits the coagulation pathway wherein prothrombin is converted to thrombin, leading to the production of fibrin from fibrinogen. Fibrin then forms the blood clot. The drug's anticoagulation activity is due to its binding to a protease inhibitor, antithrombin II (ATII) (a pentasaccharide sequence). Heparin induces a conformational change to the binding site of ATII.^13^^,^^14^ The conformational change allows for the reactive site to change, enabling the clotting enzyme to bind to ATII. Thus, the clotting enzyme is prevented from adhering to the surface of the wound, inhibiting coagulation of red blood cells.^13^^,^^14^ Heparin and the fractionated heparin (enoxaparin) must be given by injection. New orally active anticoagulants have recently been developed to interfere with the clotting system by binding directly or indirectly to Factor Xa (dabigatran, rivaroxaban, apixaban, and edoxaban) and blocking the conversion of prothrombin to thrombin.

These new products are easier to manage for patients because they can be taken orally and because their predictable effects do not require blood test monitoring of the original drugs. The fact that heparin can be easily injected intraperitoneally makes it preferable in the rat model to an oral medication. The anticoagulation these new oral drugs produce is similar to that produced by heparin and therefore the heparin model remains suitable for this assessment.

The initial step before proceeding to this study was to confirm that standard rodent dosing of heparin at 800 U/kg intraperitoneally would provide suitable anticoagulation in our rat wounding model.^11^^,^^12^ Figure 1 displays results of the preliminary experiment, indicating that heparin does indeed prevent the coagulation of blood to a suitable degree in this model.

### Mode of action of chitosan

Patients taking anticoagulants have prolonged blood coagulation that results in impairment of the healing process. Our quantified results demonstrate the effectiveness of the chitosan-based dressing immediately upon application (significant during the first 3 minutes).

The mechanism of action of chitosan is still elusive. On the basis of available data, a plausible explanation is that the negatively charged red blood cell membranes allow the protonated amine groups on the chitosan to attract these red blood cells, resulting in hemagglutination.^15^^,^^16^ A previous study has shown that chitosan encourages platelet adhesion and activation via protein adsorption and orientation, which, in turn, affect cellular responses to proteins.^17^ Chitosan's ability to enhance platelet aggregation by adsorbing plasma proteins and fibrinogen can be an important feature of its action.^4^ Chitosan subsequently leads to more platelet aggregation because it enhances the expression of glycoprotein IIb/IIIa (GPIIb/IIIa), a glycoprotein.^4^ Chitosan signals for thrombin, clotting promoter, to form, which then stimulates for the activation of GPIIb/IIa.^18^ Fibrinogen and GPIIb/IIIa bind to the platelet and activate it; inhibiting the GPIIb/IIIa receptor prevents the binding of fibrinogen to the platelets.^18^ Chitosan-based dressing (Opticell) has an efficient mechanism for controlling bleeding. However, the true mechanism of action of chitosan is yet to be fully understood.

In summary, the results from the *bleeding timeline* indicate that the chitosan-based dressing is superior to standard gauze dressing for control of bleeding from excisional wounds in heparinized rats. Bleeding was reduced immediately in the first 3 minutes after application of the chitosan dressing; the total bleeding group showed a greater than 50% reduction in blood loss in the chitosan-based dressing group compared with the standard gauze group. Chitosan dressing has hemostatic effects that could be a useful tool to control bleeding associated with wound debridement in patients with a bleeding diathesis.

## INNOVATION

Chitosan-based dressings are a novel dressing in wound care with the ability to accelerate clotting time. Used on full-thickness wounds, chitosan dressing provides a safe and efficient method for hemostasis in excisional wounds in an animal model utilizing heparin anticoagulation.

## KEY FINDINGS

- Wounds treated with chitosan dressing showed significantly less blood loss than wounds treated with standard gauze.
- The effect of chitosan on wound hemostasis occurred primarily in the first 3 minutes.
- Opticell, the chitosan-based dressing, has hemostasis properties that may be a suitable option for patients taking anticoagulants.
